# Demonstration of epinephrine autoinjectors (EpiPen and Anapen) by pharmacists in a randomised, simulated patient assessment: acceptable, but room for improvement

**DOI:** 10.1186/1710-1492-10-49

**Published:** 2014-09-19

**Authors:** Sandra M Salter, Richard Loh, Frank M Sanfilippo, Rhonda M Clifford

**Affiliations:** Pharmacy Program, Centre for Optimization of Medicines, School of Medicine and Pharmacology, The University of Western Australia, M315, 35 Stirling Highway, Crawley, WA 6009 Australia; The Australasian Society of Clinical Allergy and Immunology (ASCIA), Sydney, NSW Australia; School of Paediatrics and Child Health, The University of Western Australia, Crawley, WA Australia; School of Population Health, The University of Western Australia, Crawley, WA Australia

**Keywords:** Primary care, Pharmacy practice, Simulated patient, Anaphylaxis, EpiPen, Anapen, Technique, Self-injectable epinephrine, Adrenaline

## Abstract

**Background:**

Successful treatment of anaphylaxis in the community relies on early and correct use of epinephrine autoinjectors. Community pharmacists supply these devices and have a crucial role teaching patients how to use them. Supply of epinephrine autoinjectors in Australia increased 70-fold in the past decade. New EpiPen and Anapen autoinjectors were launched in Australia in 2011 and 2012, with the potential to cause confusion. However there is no information about how pharmacists demonstrate epinephrine autoinjectors to patients. Therefore the aim of this study was to assess real-world community pharmacist demonstrations of EpiPen and Anapen. We also sought to identify consultation-based predictors of accurate demonstration.

**Methods:**

Demonstration accuracy was assessed in simulated patient visits to 300 randomly selected pharmacies. Pharmacists were asked by the simulated patient how to use original EpiPen, new-look EpiPen or Anapen, and assessed against the relevant Australasian Society of Clinical Immunology and Allergy (ASCIA) Action Plan for Anaphylaxis. Other anaphylaxis advice provided by the pharmacist was also recorded. Accuracy was analysed descriptively. Binary logistic regression was used to identify predictors of accurate demonstration.

**Results:**

All 300 pharmacies were visited. Of 250 pharmacist demonstrations, 46 (18.4%) accurately demonstrated all four steps on ASCIA Action Plan. Failure to state ‘do not touch the needle’ (74.8%) or ‘massage injection site’ (68.8%) reduced accuracy. However 163 (65.2%) accurately demonstrated the three steps required to inject epinephrine (no difference by device, p = 0.15). Associations with accurate demonstration were: checking if the patient had an anaphylaxis action plan (odds ratio, OR = 16.1; 95% CI: 3.86-67.3); stating to call an ambulance after use (OR = 4.0; 95% CI: 1.44-11.1); or explaining side effects of epinephrine (OR = 4.5; 95% CI: 1.48-13.4).

**Conclusions:**

It is critical that anaphylaxis patients know how to use their prescribed epinephrine autoinjector correctly. Pharmacists have acceptable rates of EpiPen and Anapen demonstration accuracy, although more is needed to improve this. Those who pay attention to the need for action plans, emergency care after epinephrine use, and informing patients about the side effects of epinephrine may have better knowledge about anaphylaxis, and in turn significantly improve demonstration accuracy.

## Background

Anaphylaxis is a severe, progressive, allergic reaction that is rapid in onset and can cause death [1]. Anaphylaxis in the community is common, and increasing as severe allergies to food and insect venom rise [2–5]. Early treatment with epinephrine is essential to reduce mortality [3, 6–10]. However, in the community setting, this can represent a potentially deadly challenge for patients without immediate access to a healthcare professional. Epinephrine autoinjectors are frequently prescribed to anaphylaxis patients to enable rapid first aid before medical attention is sought [8, 9, 11]. Although not universally available, these devices exist in Australia, Canada, the United States, Europe, the United Kingdom, Asia, Africa, South America, and the Middle East [12].

In Australia, epinephrine autoinjectors (EpiPen and Anapen devices) may be obtained with or without a physician’s prescription [13, 14]. Devices supplied on prescription are subsidised through the Medicare Australia Pharmaceutical Benefits Scheme (PBS). Supply is restricted to patients who have experienced anaphylaxis, or who have been declared high-risk for anaphylaxis, by a specialist allergist, pediatrician, emergency room physician or respiratory physician. Before the listing of Anapen in 2010, EpiPen was the sole device available in Australia. A new-look EpiPen became available in 2011, and for a period all three devices were sold (original EpiPen, new-look EpiPen, Anapen). In 2003, PBS funding for epinephrine autoinjectors totalled AU$188,000. In 2013, this had risen to nearly AU$13 million; almost a 70-fold increase [15]. All of these devices were prescribed by physicians, and supplied to patients by pharmacists in community pharmacies.

It is well established that teaching patients how and when to use their autoinjector is central to sound anaphylaxis preparedness and management [3, 8, 10, 11, 16]. Alongside an understanding of the importance of timely injection is the need for correct injection technique. Erroneous injection, typically to a digit or hand, results in a lost dose of epinephrine for the patient (a potentially fatal error), as well as injury to the caregiver [17, 18]. Vigilance in training and reminding patients on correct the use of epinephrine is crucial to prevent anaphylaxis deaths. Physicians provide this training during consultations, although when assessed they have been shown to be poor demonstrators of autoinjectors [19–22]. Patients have been shown to be inconsistent with recall, and need to be regularly reminded how to use their device [20, 22–27].

Pharmacists are an important link for interdisciplinary care between physician and patient. As pharmacists see patients every time an epinephrine autoinjector is supplied, they have a unique role in teaching them how to use their device. Yet there is little research evaluating this. Studies in this area are limited to evaluation of EpiPen demonstration rates and open assessment of EpiPen demonstration steps [19, 28]. There is no blinded research assessing real-world epinephrine autoinjector technique in pharmacists, patients or other health professionals. Although Anapen is available in the United Kingdom, Europe and Australia, there is no research evaluating its demonstration by pharmacists, and few evaluations in other groups [23, 27].

Our primary aim was to investigate how accurately Australian community pharmacists demonstrated epinephrine autoinjectors to patients under real-world conditions. Pharmacists are routinely exposed to these devices, therefore we hypothesized they would have high rates of demonstration accuracy. Secondly, we considered that changes in epinephrine autoinjector availability in Australia (in 2010–2011) would cause confusion, and hypothesized that demonstration accuracy would vary between devices. In this instance we expected pharmacists would be most familiar with original EpiPen (as for more than 10 years this was the only device available in Australia); but not familiar with Anapen (a new device with different administration technique). Since new-look EpiPen was replacing original EpiPen (and they have similar technique), we expected accuracy to be similar between these devices, and higher in both compared to Anapen. Finally, we sought to identify predictors of accurate demonstration based on the features of the consultation with the pharmacist.

## Methods

We conducted a randomised, cross-sectional, simulated patient study of community pharmacist practice in Perth, Australia, from April-May 2012. Approval for the study was received from The University of Western Australia Human Research Ethics Committee in March 2012 (Approval number RA/4/1/5440). A random sample of 300 pharmacies (located within a 20 km radius of the Perth Central Business District, and listed on the Pharmacy Registration Board of Western Australia Premises Register [13]) was selected using a random numbers generator [29]. Pharmacies were randomly assigned to original EpiPen, new-look EpiPen or Anapen groups. Where the researcher recognised the pharmacist or any other staff member on duty, the visit was abandoned and the pharmacy excluded.

Devices were allocated to researchers randomly. Original EpiPen was allocated to a female Master of Pharmacy student, aged 20–25 years. New-look EpiPen was allocated to a male Master of Pharmacy student, aged 20–25 years. Anapen was allocated to an experienced simulated patient actor (female, aged 40–45 years). At each visit the researcher enacted a scenario of a patient who had experienced their first episode of anaphylaxis ‘one week ago’. Researchers carried two of the same unmarked, epinephrine autoinjector devices. All devices were new and within their expiry date, and replenished as required to ensure they appeared new for each pharmacy visit. At the pharmacy, the researcher asked for the pharmacist on duty, before showing their device and asking how to use it. Immediately after the visit (away from the premises) the researcher completed a data collection tool and subsequently entered data into a database [Microsoft Excel, Microsoft Corporation, Redmond, United States of America].

Prior to the study, we assessed the usability of the scenario during a full-day training and evaluation session. Researchers practised the role of the simulated patient, and learnt a ‘script’ so that all opening scenario statements and responses to pharmacist questions were the same for each researcher. Questions that might be asked by the pharmacist were anticipated and responses practised during the training session. If a pharmacist asked any question beyond those anticipated and for which responses had been pre-prepared, researchers answered either “I can’t remember” or “I don’t know”. EpiPen and Anapen trainers were used to teach researchers correct device demonstration, and device technique assessed to ensure accuracy.

The data collection tool captured demographic variables (broadly: pharmacy environment, pharmacist age group and gender), and self-injectable epinephrine variables (broadly: materials used for demonstration, use of references, steps used in demonstration, errors or omissions in demonstration and other advice provided). Prior to use, the tool was evaluated for face validity in a group of ten pharmacists and evaluated for usability in a round-table discussion during the training session. The scenario and data collection tool were piloted in a random sample of 9 pharmacies (3 per device). The scenario remained unchanged. Minor changes to the tool were made prior to the main study. Pharmacies visited in the pilot were not included in the final analysis. During the study, an independent auditor cross-checked a random sample of 30 completed tools against data entered in the database. The proportion of records in disagreement was 0.27%. Data in disagreement were corrected in the database prior to analysis.

We did not seek ethics approval to conduct concealed video or audio recordings in this study because demonstration is a moving visual and tactile task that is difficult to record covertly. In addition, audio recordings would not have been able to identify important features of the demonstration such as order of removal of safety caps, positioning of thumb, selection of location for injection and most importantly, which end of the device was shown as the needle end.

Accurate demonstration of epinephrine autoinjectors was defined as one that fulfilled all steps listed on the relevant Australasian Society of Clinical Immunology and Allergy (ASCIA) Action Plan for Anaphylaxis [30]; (Table 1). Errors and omissions in demonstration were recorded, along with materials used for demonstration and any additional advice provided by the pharmacist.

**Table 1:** **Steps for accurate demonstration of epinephrine autoinjectors** ^*^

	Original EpiPen	New-look EpiPen	Anapen
Step 1: Remove safety caps	Form a fist around EpiPen and remove gray safety cap	Form a fist around EpiPen and pull off blue safety release	1. Pull off black needle shield
			2. Pull off gray safety cap from red button
Step 2: Place against thigh	Place black end against outer mid thigh (with or without clothing)	Place orange end against outer mid thigh (with or without clothing)	Place needle end firmly against outer mid-thigh (with or without clothing)
Step 3: Push and inject	Push down hard until a click is heard or felt and hold in place for 10 seconds	Push down hard until a click is heard or felt and hold in place for 10 seconds	Press red button so it clicks and hold in place for 10 seconds.
Step 4: Remove and massage site	Remove EpiPen and do not touch needle. Massage injection site for 10 seconds	Remove EpiPen. Massage injection site for 10 seconds.	Remove Anapen and do not touch needle. Massage injection site for 10 seconds.

^*^Steps as listed on the relevant ASCIA Action Plan for Anaphylaxis at the time of this research [30].

### Analysis

All analyses were performed using SPSS v21 [IBM, New York, United States of America], and statistical tests reported as two-sided p-values at the 5% level of significance. Data are presented as frequencies, with associations tested using the Pearson chi-squared test or Fisher’s exact test. Compounding pharmacies and private hospital dispensaries were excluded from analysis as they may not routinely supply epinephrine autoinjectors or be directly accessible by patients.

Binary logistic regression was performed to identify consultation-specific predictors of accurate EpiPen and Anapen demonstration. Potential predictors in the model were device type, age, gender, use of references, and general anaphylaxis and device-specific information provided by the pharmacist. Recognising that consultations with pharmacists may vary from brief to extended interactions (and thus to assess the impact of predictor variables independently and collectively), we conducted both single variable and multi variable (adjusted) logistic regression analyses. Odds ratios and 95% confidence intervals for each predictor were obtained.

## Results

We visited all 300 pharmacies randomised to the study. We excluded 34 pharmacies (pilot study n = 9, known pharmacist n = 9, private hospital dispensary n = 8, premises vacant n = 4, compounding pharmacy n = 3, pharmacist not on premises n = 1). Hence, 266 (89%) of the visits were included in the final analysis. Despite randomisation, there was significant variability in the type/location of pharmacies visited and estimated pharmacist age, between EpiPen and Anapen groups. There was no difference in pharmacist gender between groups (slightly more were female: n = 155; 58.3%, p = 0.94); Table 2.

**Table 2:** **Pharmacy and pharmacist characteristics (count and %)**

Characteristic	Original EpiPen	New-look EpiPen	Anapen	Total	***P***value ^*^
	n = 87 (32.7)	n = 92 (34.6)	n = 87 (32.7)	n = 266 (100)	
**Pharmacies**					
**Pharmacy type**					0.04
Independent	43 (49.5)	47 (51.1)	36 (41.4)	126 (47.4)	
Chain	31 (35.6)	40 (43.5)	46 (52.9)	117 (44)	
Discount/warehouse	13 (14.9)	5 (5.4)	5 (5.7)	23 (8.6)	
**Pharmacy location**					0.01
Street	52 (59.8)	49 (53.3)	30 (34.5)	131 (49.2)	
Medical centre	8 (9.2)	6 (6.5)	13 (14.9)	27 (10.2)	
Shopping centre	27 (31)	37 (40.2)	44 (50.6)	108 (39.9)	
**Pharmacists**					
**Gender**					0.94
Male	35 (40.2)	39 (42.4)	37 (42.5)	111 (41.7)	
**Estimated age (years)**					0.03
20-30	35 (40.2)	46 (50)	25 (28.7)	106 (39.8)	
31-40	32 (36.8)	20 (21.7)	34 (39.1)	86 (32.3)	
41-50	11 (12.6)	18 (19.6)	22 (25.3)	51 (19.2)	
51+	9 (10.3)	8 (8.7)	6 (6.9)	23 (8.6)	

^*^Pearson chi-squared p-value for comparison of demographic variable categories across groups.

### Demonstration accuracy

Of the 266 pharmacists asked to demonstrate a device, 16 (6%) refused (4 for original EpiPen, 5 for new-look EpiPen and 7 for Anapen). Overall, 46/250 (18.4%) pharmacists who agreed to demonstrate the device accurately demonstrated all four steps of the relevant ASCIA Action Plan (significantly fewer for original EpiPen than new-look EpiPen or Anapen, p = 0.04, see Table 2). Overall, 222 (88.8%) pharmacists correctly demonstrated removal of safety caps (step 1), and 240 (96.0%) correctly demonstrated placement of the device against the mid-anterolateral thigh (step 2). Furthermore, 182 (72.8%) pharmacists correctly demonstrated how to inject (step 3). However, only 52 (20.8%) pharmacists correctly advised what to do with the device after injection (step 4). Considering the first 3 steps as those integral to receiving a dose of epinephrine, a total of 163/250 (65.2%) pharmacists completed these correctly (Table 3), with no difference between device groups (p = 0.15).

**Table 3:** **Accuracy of self-injectable epinephrine device demonstration (count and %)**

Demonstration performed	Original EpiPen	New-look EpiPen	Anapen	Total	***P***value ^*^
	(n = 83)	(n = 87)	(n = 80)	(n = 250)	
Step 1	69 (83.1)	76 (87.4)	75 (93.8)^a^	222 (86.8)	0.11
			78 (97.5)^b^		
Step 2	80 (96.4)	84 (96.6)	76 (95)	240 (96.0)	0.86
Step 3	62 (74.7)	59 (67.8)	61 (76.3)	182 (72.8)	0.42
Step 4	8 (9.6)	26 (29.9)	18 (22.5)	52 (20.8)	0.005
Steps 1-3	50 (60.2)	54 (62.1)	59 (73.8)	163 (65.2)	0.15
Steps 1-4	8 (9.6)	21 (24.1)	17 (21.3)	46 (18.4)	0.04

^*^Pearson chi-squared p-value for comparison of demonstration accuracy across groups. Step 1: Remove safety caps; Step 2: Place against mid-anterolateral thigh; Step 3: Push down hard/press red button to inject and hold for 10 seconds; Step 4: Remove device, avoid needle, massage site. ^a^Remove black needle shield. ^b^Remove gray safety cap from red button (Anapen only).

There was no difference in demonstration accuracy based on pharmacy type (p = 0.29 for comparison of 4-step accuracy, and p = 0.42 for comparison of 3-step accuracy; across independent, chain and discount pharmacies). Similarly, accuracy did not differ by pharmacy location (p = 0.89 for comparison of 4-step accuracy, and p = 0.86 for comparison of 3-step accuracy; across street, shopping centre, and medical centre locations).

### Demonstration errors

**Figure 1:**
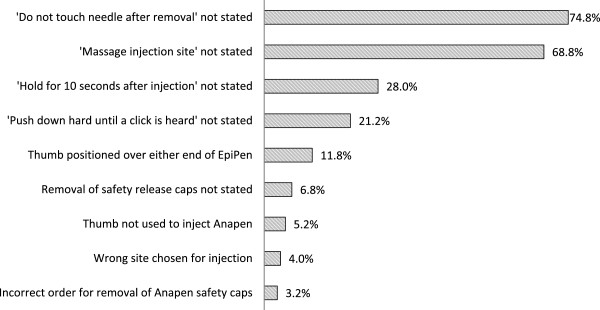
**Errors and omissions in epinephrine autoinjector device demonstration by pharmacists.**

The most frequent errors in demonstration were failure to state ‘do not touch the needle’ after injecting original EpiPen or Anapen (n = 122/163, 74.8%), or ‘massage injection site after use’ (n = 172/250, 68.8%); Figure 1. Other common errors included failure to state ‘hold in place for 10 seconds’ after injection (n = 70/250, 28%); or ‘push down hard/press the red button until a click is heard’ (n = 53/250, 21.2%). Incorrect positioning of the thumb over either end of the EpiPen was observed in 20/170 (11.8%) pharmacists.

### Demonstration materials and anaphylaxis advice

Significantly more pharmacists in the Anapen group demonstrated using the researcher’s live device (82.5% compared to 59.4% for EpiPen, p < 0.001, see Table 4). Few pharmacists used their own trainer devices (3.6% for original EpiPen, 24.1% for new-look EpiPen and 6.3% for Anapen) with the most use occurring in the new-look EpiPen group, p < 0.001. Over half (58.4%) of the pharmacists consulted reference materials including books, websites and the device itself before attempting to demonstrate, with 80-90% referring the researcher to the instructions on their device during demonstration. Of those consulting reference materials, 25/146 (17.1%) proceeded to accurately demonstrate the device (1 for original EpiPen, 10 for new-look EpiPen and 14 for Anapen; p = 0.002 for group comparison). However, 96/146 (65.8%) correctly demonstrated steps 1–3 (26 for original EpiPen, 33 for new-look EpiPen and 37 for Anapen; p = 0.04 for group comparison).

**Table 4:** **Materials used by pharmacists in self- injectable epinephrine device demonstration (count and %)**

	Original EpiPen	New-look EpiPen	Anapen	Total	***P***value ^*^
	(n = 83)	(n = 87)	(n = 80)	(n = 250)	
Researcher’s live device	50 (60.2)	51 (58.6)	66 (82.5)	167 (66.8)	<0.001
Pharmacist’s trainer device	3 (3.6)	21 (24.1)	5 (6.3)	29 (11.6)	<0.001
Consulted reference materials before demonstration	44 (53.0)	56 (64.4)	46 (57.5)	146 (58.4)	0.25
Showed instructions on researcher’s device	75 (90.4)	81 (93.1)	64 (80.0)	220 (88.0)	0.02

^*^Pearson chi-squared p-value for comparison of materials used for demonstration across groups.

Most pharmacists explained the signs of anaphylaxis (65.4%, see Table 5), asked if the researcher was aware of the precipitating allergen (66.9%), told the researcher to call an ambulance after using epinephrine (59.8%), and examined the expiry date of the device (49.6%). Pharmacists rarely checked if the patient had an anaphylaxis action plan (5.6%), or explained the side effects of epinephrine (7.9%). Compared to those demonstrating the EpiPens, significantly fewer pharmacists demonstrating Anapen explained the signs of anaphylaxis (p < 0.001), asked about the precipitating allergen (p = 0.001); or checked if the researcher had an anaphylaxis action plan (p = 0.02); Table 4.

**Table 5:** **Additional advice provided by pharmacists during consultations (count and %)**

Advice provided	Original EpiPen	New-look EpiPen	Anapen	Total	***P***value ^*^
	(n = 87)	(n = 92)	(n = 87)	(n = 266)	
Do you know what you reacted to?	66 (75.9)	67 (72.8)	45 (51.7)	178 (66.9)	0.001
Explain signs of anaphylaxis^a^	64 (73.6)	74 (80.4)	36 (41.4)	174 (65.4)	<0.001
Call ambulance after using epinephrine	53 (60.9)	60 (65.2)	46 (52.9)	159 (59.8)	0.23
Identify expiry date of researcher’s device	53 (60.9)	38 (41.3)	41 (47.1)	132 (49.6)	0.03
Are you seeing an allergy specialist?	6 (6.9)	16 (17.4)	30 (34.5)	52 (19.5)	<0.001
Conditions for device storage	20 (23)	16 (17.4)	15 (17.2)	51 (19.2)	0.49
Side effects of epinephrine	7 (8)	9 (9.8)	5 (5.7)	21 (7.9)	0.61
Do you have an anaphylaxis action plan?	4 (4.6)	10 (10.9)	1 (1.1)	15 (5.6)	0.02^b^

^*^Pearson chi-squared p-value for comparison of advice provided across groups.

^a^Pharmacist explained the signs of severe allergic reaction as listed on the relevant ASCIA Action Plan for Anaphylaxis at the time of this research [30].

^b^Fisher’s exact test used.

### Predictors of accurate demonstration

Odds ratios (OR) from the logistic regression models for predictors of accurate device demonstration are presented in Table 6. There were three significant predictors identified in both the simple and multiple regression analyses. Accurate demonstration (as part of the consultation) was more likely to occur when pharmacists: (i) asked about an anaphylaxis action plan (adjusted OR 16.1, 95% CI: 3.86-67.3); (ii) advised the researcher to call an ambulance after epinephrine use (adjusted OR 4.00, 95% CI: 1.44-11.1); or (iii) explained the side effects of epinephrine (adjusted OR 4.45 95% CI: 1.48-13.4). Additionally, in brief consultations where pharmacists may have provided just one piece of additional advice, simply explaining the signs of anaphylaxis or the conditions for device storage doubled the likelihood of accurate demonstration (OR 2.14 and 2.16 respectively). Age and gender did not impact on whether the pharmacist provided accurate demonstration. There was no difference in accuracy between new-look EpiPen and Anapen groups. However, those demonstrating the original EpiPen were 4.8 times less likely to do so accurately compared with the Anapen group (95% CI: 1.59-14.3; p = 0.006). Finally, the use of reference materials prior to demonstration did not impact on the likelihood of accurate demonstration.

**Table 6:** **Predictors of accurate self-injectable epinephrine demonstration by pharmacists** ^*^ **(n = 266)**

	***Simple regression models***			***Multiple regression model***		
Predictor	OR	95% CI	***P***value	Adjusted OR	95% CI	***P***value
Original EpiPen	0.64	0.35-1.19	0.16	0.21	0.07-0.63	0.006
New-look EpiPen	0.67	0.37-1.24	0.21	0.60	0.23-1.55	0.29
Anapen^a^	1			1		
Age 20-30	2.59	0.56-11.9	0.22	1.74	0.26-11.4	0.56
Age 31-40	1.87	0.39-8.95	0.43	1.59	0.24-10.4	0.63
Age 41-50	2.56	0.51-12.8	0.25	2.69	0.40-17.8	0.31
Age 51+^a^	1			1		
Male	0.70	0.36-1.36	0.30	0.80	0.35-1.79	0.58
Reference materials consulted	0.96	0.51-1.81	0.89	0.75	0.34-1.67	0.48
Do you know what you reacted to?	1.98	0.93-4.12	0.08	1.49	0.53-4.12	0.45
Are you seeing an allergy specialist?	1.59	0.76-3.34	0.22	0.52	0.19-1.43	0.20
Explain signs of anaphylaxis	2.14	1.01-4.54	0.05	0.90	0.33-2.47	0.84
Do you have an anaphylaxis action plan?	17.0	5.12-56.3	<0.001	16.1	3.86-67.3	<0.001
Call ambulance after epinephrine	5.66	2.30-13.9	<0.001	4.00	1.44-11.1	0.008
Conditions for device storage	2.16	1.05-4.45	0.04	1.80	0.72-4.51	0.21
Explain side effects of epinephrine	5.28	2.09-13.3	<0.001	4.45	1.48-13.4	0.008
Identify expiry date of device	1.74	0.91-3.32	0.10	1.08	0.48-2.43	0.86

^*^Accurate device demonstration required all steps on the relevant ASCIA Action Plan for Anaphylaxis [30] to be performed without error.

OR = odds ratio; 95% CI = 95% confidence interval.

^a^Reference level for the variable. Simple regression models assessed each predictor in a separate univariate model. The multiple regression model assessed all predictors at once (model fit: Nagelkerke R^2^ = 0.33).

## Discussion

Self-injectable epinephrine is the cornerstone of emergency management for anaphylaxis occurring in the community, and accurate administration technique is critical for successful use during acute events. This is the largest study of epinephrine autoinjector demonstration by community pharmacists, and the only study in any health profession to evaluate demonstration technique in a blinded manner. Further, this is the first study to assess differences in demonstration accuracy between original EpiPen, new-look EpiPen and Anapen.

### Main findings

Overall, 65% of pharmacists accurately demonstrated the first three steps required for epinephrine injection. However, only 18% of pharmacists performed all four steps listed on the ASCIA Action Plan for Anaphylaxis [30] with the proportion being twice as high for new-look EpiPen and Anapen than for original EpiPen. Thus despite more than 10 years of uninterrupted use of original EpiPen in Australia, pharmacists performed worse when demonstrating this device compared to the new devices. Seemingly familiarity with a device does not guarantee sound technique; rather being ‘unusual’ or ‘new’ may serve to make the user more careful.

In the broader context no other evaluation of device technique has assessed the fourth step (remove device after injection, do not touch needle, massage injection site for 10 seconds). It is possible that pharmacists considered this last step did not require explanation, as it is obvious the device needs to be removed after injection. Furthermore, the importance of massaging the site is unknown, although it may provide comfort from the puncture of the needle. Studies of epinephrine autoinjector demonstration in physicians and patients focus only on the three steps required for injection of original EpiPen. These show that 21-41% of allergy specialists [19, 22]; 11-37% of other medical practitioners [19, 21, 31, 32]; and 9-36% of patients and caregivers [25, 26] accurately performed those three steps. Therefore, against the 4-step ASCIA standard [30] pharmacists had a low proportion who demonstrated accurately, whereas in comparison with previous research (3-step evaluation), pharmacists had the highest proportions of accurate demonstrators.

Although all of the autoinjectors have the same cost and are subsidised equally on the PBS, EpiPen is more widely prescribed in Australia [15, 33]. We expected demonstration accuracy to be similar between the EpiPens but poorer in the Anapen group. Therefore it was surprising that 4-step accuracy was significantly worse for original EpiPen, and that there was no difference in 3-step accuracy between all 3 devices. Given this research was conducted in the midst of the change in device availability it is possible pharmacists had been exposed to promotional materials from the new autoinjector manufacturers, or had undertaken self-directed learning to address a perceived unfamiliarity with new-look EpiPen and Anapen.

### Features of the consultation associated with accurate demonstration

Our study found that three elements of the pharmacist’s advice showed an association with accurate device demonstration. Firstly, the odds of an accurate demonstration was 16 times higher if the pharmacist asked the researcher ‘do you have an anaphylaxis action plan?’ In Australia, patients prescribed epinephrine autoinjectors on a PBS prescription must also receive an anaphylaxis emergency management plan [33]. These plans are prepared by the specialist allergist and rarely seen by the patient’s pharmacist. Only 5.6% of pharmacists in this study asked about an action plan, and in practice less than half of all anaphylaxis patients actually have an action plan [34, 35]. Yet there is immense potential for improvement in device demonstration, patient preparedness and understanding of anaphylaxis with their use [3, 8–11, 36, 37]. Judicious use of device-specific anaphylaxis action plans in pharmacist consultations may improve demonstration accuracy (especially since the steps for demonstration are shown on the plan), while reminding patients of the need to obtain their own plan.

Advising the researcher to call an ambulance after epinephrine use was associated with a four-fold increase in the odds of an accurate demonstration, and explaining the side effects of epinephrine was associated with a 4.5 fold increase in the odds of an accurate demonstration. Although 60% of pharmacists in this study recognised the need for emergency care after epinephrine use, [3, 7–10, 38–41] less than 10% of pharmacists explained the side effects of epinephrine. Provision of medicines and other information is fundamental to professional pharmacist practice [42], and although there are benefits to the patient in providing such advice (empowerment, comfort with intended use [43, 44]), these elements cannot alone predict technical expertise or accurate autoinjector demonstration. Rather, they likely reflect a more detailed understanding of anaphylaxis and epinephrine autoinjector devices, and it is this deeper knowledge that contributes to improved autoinjector demonstration accuracy.

Predictors of accurate epinephrine device demonstration by physicians and patients relate to their experiences. Regular allergy assessment, history of severe anaphylaxis and recognition of the device are important predictors of demonstration accuracy in physicians, while practical demonstration, prior consultation with a specialist and empowerment (including independently seeking information) impact patient technique [21, 24, 43, 45]. Given this was a simulated patient study and pharmacists were unaware they were being assessed, it was not possible to measure experience as a predictor of accurate demonstration.

### Relevance of demonstration errors

Common errors in demonstration were similar to those observed in other studies [19, 21, 22, 24, 31, 32, 46]. Failure to activate the device (5-21% of pharmacists), or to hold it firmly in place for 10 seconds after injection (28%) prevents or reduces the intramuscular dose of epinephrine received. Positioning the thumb over either end of EpiPen (11.8%) may result in unintentional injection to the thumb and a lost dose for the patient. These errors are of concern: in one-third of visits the researcher would not have received epinephrine had they followed the pharmacist’s instructions.

More than two-thirds of pharmacists in our study did not inform the researcher of the fourth step: avoid the exposed needle (original EpiPen and Anapen), and massage the site after injection. These statements are clearly defined in device-specific ASCIA Action Plans for Anaphylaxis and in manufacturer information [30, 47–49], but are not printed in the instructions on either live or trainer epinephrine autoinjector devices. There is no information on the frequency or hazards of needle-stick injury after intentional epinephrine autoinjector use. While the clinical implication of failure to massage after injection is unclear, a slower rate of epinephrine absorption or injection site discomfort may be relevant. The lack of any research amongst physicians, patients or carers involving this step suggests it is not perceived to be important in device administration. Similarly, it is likely that pharmacists do not consider these ‘after-injection’ steps as relevant to receiving a dose of epinephrine. As this research is the first to evaluate the fourth step, future consideration of the need for this step in inclusion in manufacturer information, patient leaflets and anaphylaxis action plans is needed. As a minimum, consistency across information sources (written materials and autoinjector devices) should be a priority.

### Strengths and limitations

In previous research on epinephrine autoinjector demonstration, users were 2.6 times more likely to perform accurate demonstration when informed that the technique would be assessed [50]. Therefore a key strength of this study was the use of simulated patient methodology to blind pharmacists to their assessment. This technique is well described in the literature as a tool to measure true pharmacist practice, and overcomes the issues of participant bias that occurs when pharmacists know they will be evaluated [51, 52]. Further, it is common in Australia for patients to request advice over-the-counter at a pharmacy without a product sale or prescription purchase, so we did not consider the request would arouse suspicion.

However, because we solicited the pharmacist’s advice without a sale, we could not evaluate true patterns of device demonstration that may occur when an epinephrine autoinjector is actually dispensed. Moreover, the complexities of PBS subsidies and high cost of self-injectable epinephrine (currently more then AU$100 for one EpiPen or Anapen) [33] preclude such an evaluation.

As we did not record the exchange between pharmacist and simulated patient, we relied on researchers to remember the consultation with the pharmacist and acknowledge that recall bias was possible. Further we note that the use of Master of Pharmacy students as simulated patients presented the potential for bias in scenario delivery or responses to pharmacist questions, although we aimed to minimise this with predefined statements for each researcher to use. In addition, as all three simulated patients required sound knowledge of anaphylaxis and epinephrine autoinjector technique (to be able to judge and record the consultation), we cannot be sure how a lay consumer would interpret and remember the consultation. Nor did we evaluate whether the pharmacist asked the researcher to demonstrate the device back to them, and in turn assess the true understanding gained as a result of the pharmacist demonstration. This is an essential part of device training and a gap in research that should be addressed.

Finally, although we found three elements of pharmacist advice were associated with significantly higher odds of accurate demonstration (both independently and collectively), they do not predict accurate demonstration within themselves, but represent better knowledge of anaphylaxis and autoinjector technique in general.

### Implications and recommendations

This study showed pharmacists were willing to demonstrate EpiPen and Anapen devices to patients even when they had not supplied them and would not receive remuneration for the service. Two-thirds of pharmacists under the blinded real-world conditions of our study provided sound demonstration advice, compared to one-third of physicians in open evaluations [19, 31, 32]. Thus despite concerns that pharmacists are poor demonstrators [53] we may be reassured that most *can* show their patients how to inject a dose of epinephrine. For the one-third of pharmacists who failed to do so accurately, the use of device-specific anaphylaxis action plans during demonstration may prompt improved accuracy.

While we recognise the importance of an holistic approach in autoinjector device training, pharmacists are a sound option for training in the periods when physician review is not possible. Patients may wait months after diagnosis for an appointment with their allergy specialist, or only see their general practitioner when a new prescription is required to replace an expired device (every 1–2 years) [10]. Consultations may be time-restricted and patients may be overwhelmed with new information, reducing the potential for a memorable device demonstration by physicians. In-time retraining of autoinjector technique by pharmacists should be considered to raise patients’ awareness and competence with autoinjector devices.

Dealing with the errors in device technique is essential, yet may be difficult to achieve because aspects critical for epinephrine injection (such as pushing hard to inject, holding the device in place for 10 seconds, or using the correct thumb position) are not intuitive. Novel approaches should be developed for training. Beyond this training is the need to prevent errors in the high-stress environment of acute anaphylaxis. Performing device demonstrations under pressure (such as with a timer), and then evaluating patient technique similarly would be useful to prepare patients to work quickly in real emergencies.

## Conclusions

Pharmacists in Australia dispense prescriptions for epinephrine autoinjectors 70 times more often now than ten years ago [15]. Given their important role, and significant potential in anaphylaxis preparedness, it is disappointing that only 18% of them accurately demonstrated all four steps for autoinjector administration listed on the ASCIA action plan for anaphylaxis [30]. However, the fourth step is not relevant to the patient receiving a dose of epinephrine and has not been not tested in any other research. Moreover 65% of pharmacists accurately demonstrated all 3 steps required for epinephrine injection from an autoinjector. Notably, this is the best demonstration accuracy observed in any health professional group. Nonetheless there remains room for improvement. Raising awareness of the need for action plans, emergency care after epinephrine use, and informing patients about the side effects of epinephrine may prompt recall of epinephrine autoinjector technique and improve demonstration accuracy. This in turn may improve epinephrine autoinjector use in the community and save lives.
